# SIRT6 Acts as a Negative Regulator in Dengue Virus-Induced Inflammatory Response by Targeting the DNA Binding Domain of NF-κB p65

**DOI:** 10.3389/fcimb.2018.00113

**Published:** 2018-04-09

**Authors:** Pengcheng Li, Yufei Jin, Fei Qi, Fangyi Wu, Susu Luo, Yuanjiu Cheng, Ruth R. Montgomery, Feng Qian

**Affiliations:** ^1^Ministry of Education Key Laboratory of Contemporary Anthropology, School of Life Sciences, Fudan University, Shanghai, China; ^2^Institute of Biothermal Science and Technology, School of Medical Instrument and Food Engineering, University of Shanghai for Science and Technology, Shanghai, China; ^3^Program on Human Translational Immunology, Department of Internal Medicine, Yale University School of Medicine, New Haven, CT, United States

**Keywords:** SIRT6, dengue virus, proinflammatory cytokines, NF-κB p65, RLR, TLR3

## Abstract

Dengue virus (DENV) is a mosquito-borne single-stranded RNA virus causing human disease with variable severity. The production of massive inflammatory cytokines in dengue patients has been associated with dengue disease severity. However, the regulation of these inflammatory responses remains unclear. In this study, we report that SIRT6 is a negative regulator of innate immune responses during DENV infection. Silencing of *Sirt6* enhances DENV-induced proinflammatory cytokine and chemokine production. Overexpression of SIRT6 inhibits RIG-I-like receptor (RLR) and Toll-like receptor 3 (TLR3) mediated NF-κB activation. The sirtuin core domain of SIRT6 is required for the inhibition of NF-κB p65 function. SIRT6 interacts with the DNA binding domain of p65 and competes with p65 to occupy the *Il6* promoter during DENV infection. Collectively, our study demonstrates that SIRT6 negatively regulates DENV-induced inflammatory response via RLR and TLR3 signaling pathways.

## Introduction

Dengue virus (DENV) is a mosquito-transmitted single-stranded RNA virus, causing an acute systemic viral disease and is a significant public health concern (Guzman et al., 2010). An estimated 390 million individuals worldwide are infected with DENV per year, with 96 million manifesting symptoms (Bhatt et al., 2013). Although primary infection in humans typically results in mild dengue fever, secondary infection with different DENV serotypes is associated with severe symptoms which can lead to dengue hemorrhagic fever (DHF), dengue shock syndrome (DSS) and even death (Wilder-Smith and Schwartz, 2005). It is believed that the massive production of inflammatory cytokines contributes to the pathogenesis of severe dengue disease (Pang et al., 2007). Unfortunately, there are no DENV specific therapies or widely available vaccines (Martin and Hermida, 2016).

Pattern recognition receptors (PRRs) are key components of the innate immune system that recognize pathogen associated molecular patterns (PAMPs) (Chan and Gack, 2016). DENV infection is recognized by the innate immune system through retinoic acid-inducible gene-I (RIG-I)-like receptors (RLRs) and Toll like receptors (TLRs) (Tsai et al., 2009; Nasirudeen et al., 2011). These two recognition pathways lead to the activation of a series of signaling events and the induction of proinflammatory cytokines and chemokines. The cytosolic sensor RIG-I detects DENV after fusion and replication in infected cells. TLR3 recognizes dsRNA in endosomes (Green et al., 2014). Upon recognition of viral RNA, the caspase-recruitment domain of RIG-I binds to the mitochondrial antiviral signaling (MAVS) adaptor, while TLR3 interacts with the TIR domain containing adaptor TRIF. These adaptors then recruit E3 ligase TRAF6, and further activate the canonical IKKs, leading to the production of nuclear factor-κB (NF-κB) dependent proinflammatory cytokines (Kawai and Akira, 2009; Kumar et al., 2011).

NF-κB is a critical transcriptional factor that participates in the regulation of inflammatory mediators. After viral infection, NF-κB can be activated by several pathways including RLRs and TLRs mediated signaling (Rahman and McFadden, 2011). The most abundant form of NF-κB is a p50/p65 heterodimer, which associates with the NF-κB inhibitor α (IκBα). Following stimulation, IκBα is phosphorylated and then degraded. NF-κB translocates to the nucleus where it induces the expression of NF-κB-target genes (Li and Verma, 2002). NF-κB activation during DENV infection has been reported to lead to the induction of cell apoptosis and excessive inflammation (Jan et al., 2000). In a murine model, NF-κB activation induced by DENV protease overexpression resulted in development of dengue hemorrhage (Lin et al., 2014). In humans, increased levels of NF-κB regulated proinflammatory cytokine TNFα have been associated with the severity of dengue disease manifestations (Soundravally et al., 2014). Thus, excessive NF-κB activation should be tightly controlled. The negative regulatory mechanisms of NF-κB activation remain incompletely defined.

The sirtuins (SIRTs) are a family of nicotinamide adenine dinucleotide (NAD)-dependent deacetylases. Mammals contain seven sirtuins, which have different subcellular localizations, with a subset of sirtuins residing in mitochondrial (SIRT3, SIRT4 and SIRT5), cytosolic (SIRT2), or nuclear (SIRT1, SIRT6, and SIRT7) compartments (Haigis and Sinclair, 2010). SIRT6, predominantly a nuclear protein, has been increasing identified as a critical regulator in diverse physiological and pathological events including life span, DNA damage repair, glucose metabolism and cancer (Gertler and Cohen, 2013). SIRT6 is known to deacetylate lysine-9 of histone H3 at the promoter of many genes involved in glycolysis and lipid metabolism (Kim et al., 2010). SIRT6 is also known to mediate mono-ADP ribosylation of KAP1 and repress LINE1 retrotransposons (Van Meter et al., 2014). The *in vivo* studies have shown that SIRT6 prevent age-related disorders and premature aging. Transgenic overexpression of SIRT6 could extend lifespan in male mice (Kanfi et al., 2012). The phenotype of *Sirt6*^−/−^ mice is the consequence of multiorgan degeneration associated with premature aging and chronic inflammation (Mostoslavsky et al., 2006; Xiao et al., 2012). Sirtuins may play an important role in the control of inflammation through the regulation of immune gene transcription. SIRT1, one of the most widely studied nuclear sirtuin, has been shown to suppress inflammatory responses. Resveratrol, an activator of SIRT1, inhibits inflammation by targeting NF-κB signaling (Zhu et al., 2011). SIRT2 suppresses inflammatory responses in collagen-induced arthritis (Lin et al., 2013). The role of SIRT6 in antiviral innate immune responses and whether SIRT6 is involved in the regulation of DENV-induced inflammatory response is currently unknown.

Here we demonstrate that SIRT6 negatively regulates the DENV-induced inflammatory response. SIRT6 is induced upon DENV infection, and proinflammatory cytokine production to DENV is enhanced when *Sirt6* is silenced. Further studies show that SIRT6 binds to the DNA binding domain of p65 and inhibits NF-κB function.

## Materials and methods

### Cell lines, viruses, and reagents

The Raw264.7 cell line was a kind gift form Dr. Zhaojun Wang (Jiaotong University, China). HEK293T cells were obtained from Type Culture Collection of the Chinese Academy of Science. The cells were cultured at 37°C under 5% CO_2_ in DMEM or RPMI 1640 medium supplemented with 10% fetal bovine serum and antibiotics (100 units/mL penicillin and 100 μg/mL streptomycin, Invitrogen). DENV New Guinea C strain serotype 2 (DENV-2) was propagated in C6/36 cells. Low molecular weight (LMW) and high molecular weight (HMW) Poly I:C were from Invivogen. The antibodies specific to SIRT6, p65, Histone H3, Caspase 3 were from Cell Signaling Technology. Anti-HA, anti-Flag, anti-p50 and anti-GAPDH antibodies were from Proteintech. HRP-conjugated secondary antibodies were from Sungene Biotechnology.

### shRNAs and lentiviral infection

The small hairpin RNA (shRNA) target sequences were as follows: mouse *Sirt6* sh*Sirt6*-1 (5′-TCC CAA GTG TAA GAC GCA GTA-3′), sh*Sirt6*-2 (5′-GCA TGT TTC GTA TAA GTC CAA-3′), human *SIRT6* shRNA (5′-CTC CCT GGT CTC CAG CTT AAA-3′), mouse p65 shRNA (5′-GCA TGC GAT TCC GCT ATA AAT-3′) and scrambled shRNA (5′-CAA CAA GAT GAA GAG CAC CAA-3′). Lentiviruses expressing scrambled or *Sirt6* specific shRNA were used to generate control and *Sirt6*-knockdown cell lines. Lentivirus was produced by cotransfection of HEK293T cells with pLKO.1 shRNA plasmid (Addgene plasmid No. 8453), pCMV-dR8.2 dvpr packaging plasmid (Addgene plasmid No. 8455), and pCMV-VSVG envelope plasmid (Addgene plasmid No. 8454). Supernatants containing lentivirus were harvested at 36–48 h after transfection and used to infect the target cells for 24 h. Stably transduced cells were selected in media containing puromycin (5 μg/mL; Invivogen). Knockdown efficiency was analyzed by immunoblot.

### Dual-luciferase reporter assay

HEK293T cells were transfected in 24-well plates with indicated expression plasmids along with luciferase reporter plasmids pNF-κB-Luc (Stratagene), TNFa-Luc (kindly provided by Dr. Guang Yang, Jinan University, China) or pRL-TK (Promega) using Hieff Trans^TM^ transfection reagent (Yeasen). The cells were incubated at 37°C under 5% CO2 for 24 h. For experiments with ligand stimulation, cells were treated with HWM or LMW Poly I:C (10 μg /mL) for 16 h. Cells were lysed, and luciferase activities were determined with Dual-Luciferase Reporter Assay System (Wang et al., 2017).

### Quantitative PCR (qPCR) analysis

Total RNA was extracted using TRI Reagent (Sigma), and cDNA was synthesized using the Reverse Transcription Reagent Kit (Takara). Amplification was performed using SYBR Green qPCR Master Mix (Biotools) with gene-specific primers in CFX96 System (Bio-Rad). Gene expression levels were normalized to the *Actin* gene. Primer sequences used for qPCR were as follows: *mSirt6* (forward: 5′-CAG TAC GTC AGA GAC ACG GTT G-3′, reverse: 5′-GTC CAG AAT GGT GTC TCT CAG C-3′), *mIl6* (forward: 5′-TAC CAC TTC ACA AGT CGG AGG C-3′, reverse: 5′-CTC AAG TCA TCA TCG TTG TTC-3′), *mTnf*α (forward: 5′-GGT GCC TAT GTC TCA GCC TCT T-3′, reverse: 5′-GCC TAG AAC TGA TGA GAG GGA G-3′), *mCcl2* (forward: 5′-GCT ACA AGA GGA TCA CCA GCA G-3′, reverse: 5′-GTC TGG ACC CAT TCC TTC TTG G-3′), *mCcl5* (forward: 5′-CCT GCT GCT TTG CCT ACC TCT C-3′, reverse: 5′-ACA CAC TTG GCG GTT CCT TCG A-3′), *mActin* (forward: 5′-CAT TGC TGA CAG GAT GCA GAA GG-3′, reverse: 5′-TGC TGA AGG TGA CAT GAG G-3′). *hIL6* (forward: 5′-AGA CAG CCA CTC ACC TCT TCA G-3′, reverse: 5′-TTC TGC CAG TGC CTC TTT GCT G-3′), *hTNFA* (forward: 5′-CTC TTC TGC CTG CTG CAC TTT G-3′, reverse: 5′-ATG GGC TAC AGG CTT GTC ACT C-3′), *hCCL2* (forward: 5′-GCT ACA AGA GGA TCA CCA GCA G-3′, reverse: 5′-GTC TGG ACC CAT TCC TTC TTG G-3′), *hCCL5* (forward: 5′-CCT GCT GCT TTG CCT ACA TTG C-3′, reverse: 5′-ACA CAC TTG GCG GTT CTT TCG G-3′), *hACTIN* (forward: 5′-AGA TCA TGT TTG AGA CCT TCA ACA C-3′, reverse: 5′-GGA GCA ATG ATC TTG ATC TTC ATT G-3′). *DENV E* (forward: 5′-CAT TCC AAG TGA GAA TCT CTT TGT CA-3′, reverse: 5′-CAG ATC TCT GAT GAA TAA CCA ACG-3′).

### Cytokine ELISA measurements

Cell supernatants were harvested from DENV infected cells. Supernatants were centrifuged for 5 min at 3,000 g to remove cellular debris and were stored at −80°C before analysis. Cytokines were quantified using ELISA MAX^TM^ Deluxe kit (Biolegend) according to the manufacturer's instructions.

### Coimmunoprecipitation (CoIP) and immunoblot analysis

HEK293T cells transfected with expression plasmids or Raw264.7 cells were harvested using RIPA III lysis buffer containing protease inhibitor cocktail (Biotechwell). Whole cell extracts were incubated overnight at 4°C with indicated antibodies followed by incubation with protein A/G agarose beads (Santa Cruz) for 2 h at 4°C. The beads were washed five times with lysis buffer and proteins eluted by boiling for 5 min in SDS sample buffer.

For immunoblot analysis, cell lysates were subjected to SDS-PAGE and transferred onto PVDF membranes (Thermo Fisher). Immunoblots were probed with indicated antibodies and developed using NcmECL Ultra Reagent (NCM Biotech).

### Immunofluorescence microscopy

Poly I:C treated and untreated cells were fixed, permeabilized in 4% PFA/0.2 Triton-X for 20 min and blocked in PBS/10% FBS for 1 h. Cells were labeled with mouse anti-Flag and rabbit anti-HA antibodies, and detected by Alexa 488 anti-mouse and Alexa 546 anti-rabbit antibodies (Invitrogen). Images were collected using an inverted fluorescence microscope (Leica).

### Chromatin immunoprecipitation

Cells were fixed with 1% formaldehyde and quenched with glycine. Purified chromatin was sonicated to ~500 bp using the Ultrasonic Processing (SONICS) and incubated with the indicated antibodies. DNA-protein complexes were immunoprecipitated by protein A/G agarose, followed by reverse crosslinking processes. The DNA was then purified and quantified by qPCR. PCR primer sequences for promoter regions were as follows: *Il6*-CHIP (forward: 5′-GCA GTG GGA TCA GCA CTA AC-3′, reverse: 5′-GGT GGG TAA AGT GGG TGA AG-3′), *Tnfa*-CHIP (forward: 5′-GGA GAT TCC TTG ATG CCT GG-3′, reverse: 5′-GCT CTC ATT CAA CCC TCG GA-3′).

### Statistical analysis

Statistical significance was determined by an unpaired Student's *t*-test with GraphPad Prism. Data were presented as mean ± SEM of three independent experiments. Values of *p* < 0.05 were considered statistically significant.

## Results

### SIRT6 negatively regulates DENV-induced proinflammatory cytokine and chemokine production

**Figure 1:**
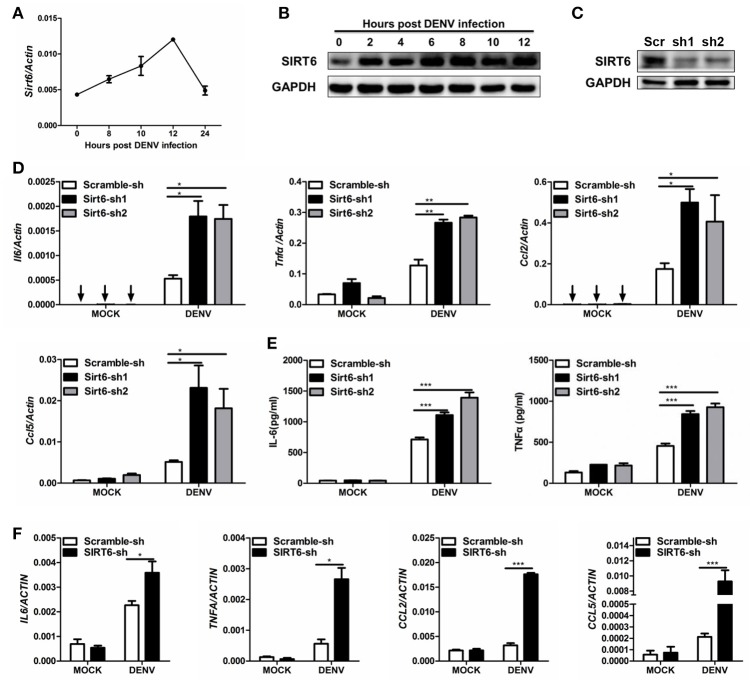
SIRT6 negatively regulates DENV-induced proinflammatory cytokine and chemokine production. Quantification of **(A)** *Sirt6* mRNA levels by qPCR, and **(B)** SIRT6 protein expression by immunoblot in Raw264.7 cells after DENV infection (MOI = 1). **(C)** Immunoblot analysis of SIRT6 expression in Raw264.7 cells stably expressing shRNA against *Sirt6*. Quantification of **(D)** indicated cytokine and chemokine mRNA levels by qPCR and **(E)** secreted IL-6 and TNFα levels by ELISA from Raw264.7 cells stably expressing either scrambled shRNA or *Sirt6*-targeting shRNA after DENV infection for 12 h. **(F)** Quantification of indicated cytokine and chemokine mRNA levels by qPCR from HEK293T cells stably expressing either scrambled shRNA or *SIRT6*-targeting shRNA after DENV infection for 24 h. Data shown are the mean ± SEM; ^*^p < 0.05, ^**^p < 0.01, ^***^p < 0.001. Representative results are from at least three independent experiments.

Macrophages and mononuclear phagocytes are the primary targets of dengue virus infection (Chen and Wang, 2002). We examined the expression pattern of SIRT6 in murine Raw264.7 macrophages upon DENV infection. The mRNA level of *Sirt6* was significantly increased after DENV infection within 12 h (Figure 1A). Immunoblot further confirmed the upregulation of SIRT6 protein expression in DENV infected cells (Figure 1B).

Macrophages are the major source of proinflammatory cytokines after DENV infection. To investigate the potential role of *Sirt6* in DENV-induced inflammatory response, we designed small hairpin RNA (shRNA) that targeted two sites of *Sirt6* and generated *Sirt6*-silenced Raw264.7 cells. Endogenous SIRT6 was silenced efficiently as quantified by immunoblot analysis (Figure 1C). We used qPCR to quantify mRNA expression levels of proinflammatory cytokine and chemokine in *Sirt6*-silenced cells. Notably, silencing of *Sirt6* significantly increased the transcription level of proinflammatory cytokines *Il6 and Tnf*α as well as chemokines *Ccl2* and *Ccl5* after DENV infection (Figure 1D). We further measured protein production of IL-6 and TNFα in *Sirt6*-silenced cells after DENV infection. In agreement with these findings, these cells produced greater amounts of IL-6 and TNFα protein than scrambled shRNA-treated cells (Figure 1E). To determine whether the function of SIRT6 is conserved across species, we generated *SIRT6*-silenced HEK293T human embryonic kidney cells. Similar to the data obtained with Raw264.7 cells, we observed that knockdown of *SIRT6* in HEK293T increased DENV-induced expression levels of *IL6, TNFA, CCL2*, and *CCL5* (Figure 1F). Moreover, DENV replication was reduced when *SIRT6* was silenced (Figure S1). These results suggest that SIRT6 is involved in the regulation of DENV-induced proinflammatory cytokine and chemokine production.

### SIRT6 inhibits RLR-mediated inflammatory response

**Figure 2:**
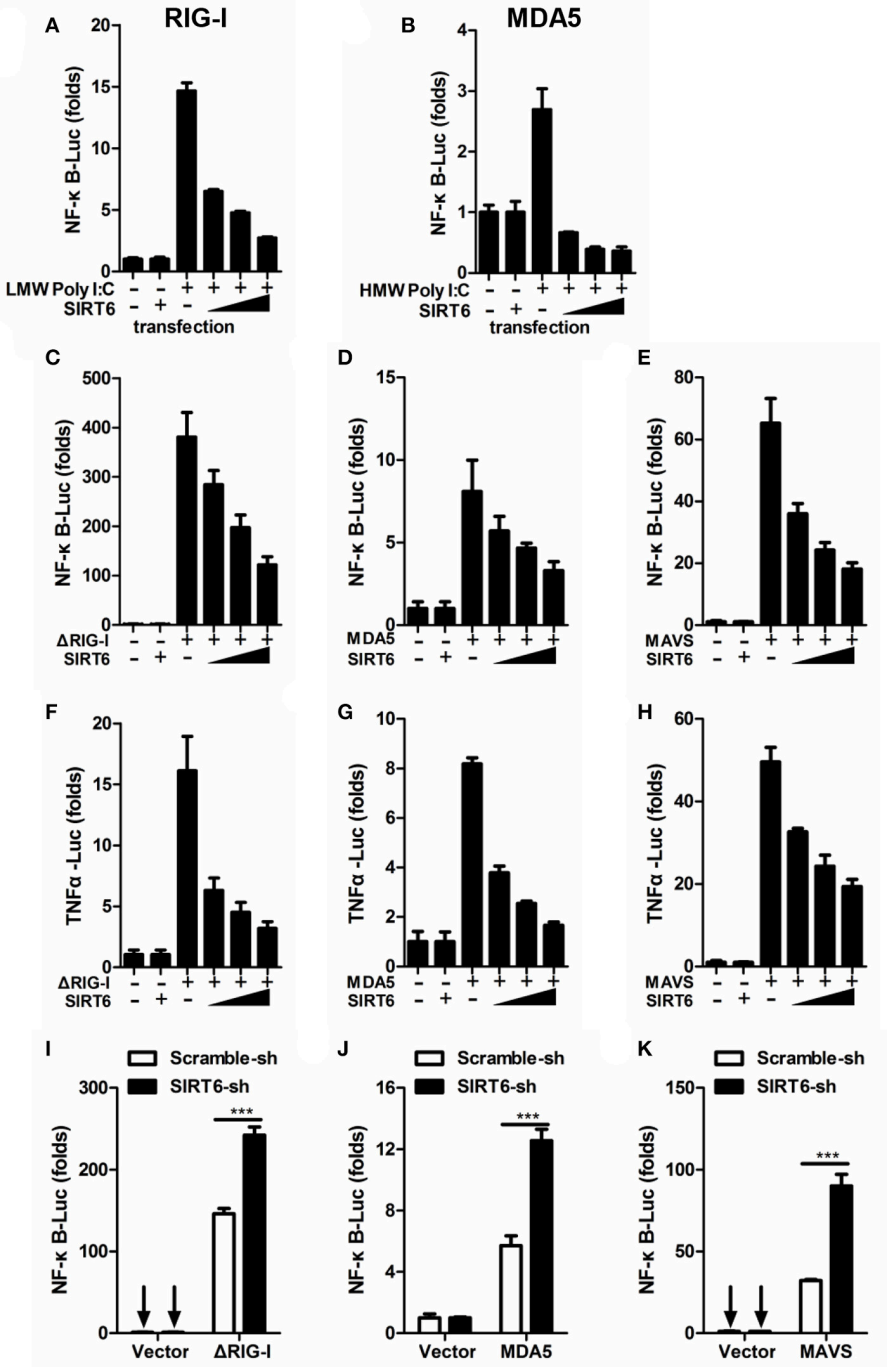
SIRT6 inhibits RLR-mediated inflammatory response. **(A,B)** Quantification of NF-κB promoter activity of HEK293T cells transfected with either an empty vector or increasing amounts of SIRT6 plasmid and treated with LMW Poly I:C or HMW Poly I:C. Quantification of **(C–E)** NF-κB promoter activity and **(F–H)** TNFα promoter activity of HEK293T cells expressing ΔRIG-I, MDA5, or MAVS, together with increasing amounts of SIRT6. **(I–K)** Quantification of NF-κB promoter activity in scrambled shRNA or *SIRT6* shRNA treated HEK293T cells transfected with either an empty vector or ΔRIG-I, MDA5, MAVS plasmids. Data shown are the mean ± SEM; ^***^p < 0.001. Representative results are from at least three independent experiments.

The RLR pathway is a well-characterized pathway initiating antiviral innate immune responses (Loo and Gale, 2011). The key transcription factor NF-κB contributes to the production of proinflammatory cytokines in RLR-mediated signal transduction. We examined whether SIRT6 regulated virus-induced inflammatory reactions through the RIG-I/MDA5 pathway. By transfection of a synthetic analog of double-stranded RNA (agonist of RLR pathway), we initially tested the effect of SIRT6 on RLR-dependent NF-κB activation. We found that SIRT6 significantly inhibited activation of NF-κB promoter in HEK293T cells transfected with either low-molecular-weight (LMW) Poly I:C (RIG-I activator) or high-molecular-weight (HMW) Poly I:C (MDA5 activator) (Figures 2A,B).

To confirm the inhibitory role of SIRT6 in RLR signaling, we tested the effect of SIRT6 on upstream components of the RLR signaling pathway in their activation of NF-κB and TNFa promoters through reporter assay. We transfected HEK293T cells with expression plasmids encoding the RLR signaling molecules ΔRIG-I (active form of RIG-I), MDA5 or MAVS (adaptor molecule), together with increasing amounts of SIRT6 and luciferase reporter constructs driven by the transcription factor NF-κB or TNFα promoter. SIRT6 overexpression significantly decreased ΔRIG-I, MDA5, and MAVS-induced NF-κB activation (Figures 2C–E, Figure S2). Furthermore, SIRT6 also inhibited ΔRIG-I, MDA5, and MAVS-induced TNFα promoter activation in a dose-dependent manner (Figures 2F–H). In contrast, silencing of *SIRT6* enhanced the activation of NF-κB by these RLR signaling molecules (Figures 2I–K). The expression level of NF-κB responsive gene *Il6* was increased in *Sirt6-*knockdown cells after SeV (agonist of the RLR signaling) infection (Figure S3A). Together, these data suggest that SIRT6 is a negative regulator for the RLR signaling pathway and inhibits RLR-mediated NF-κB activation.

### SIRT6 inhibits TLR3-mediated inflammatory response

**Figure 3:**
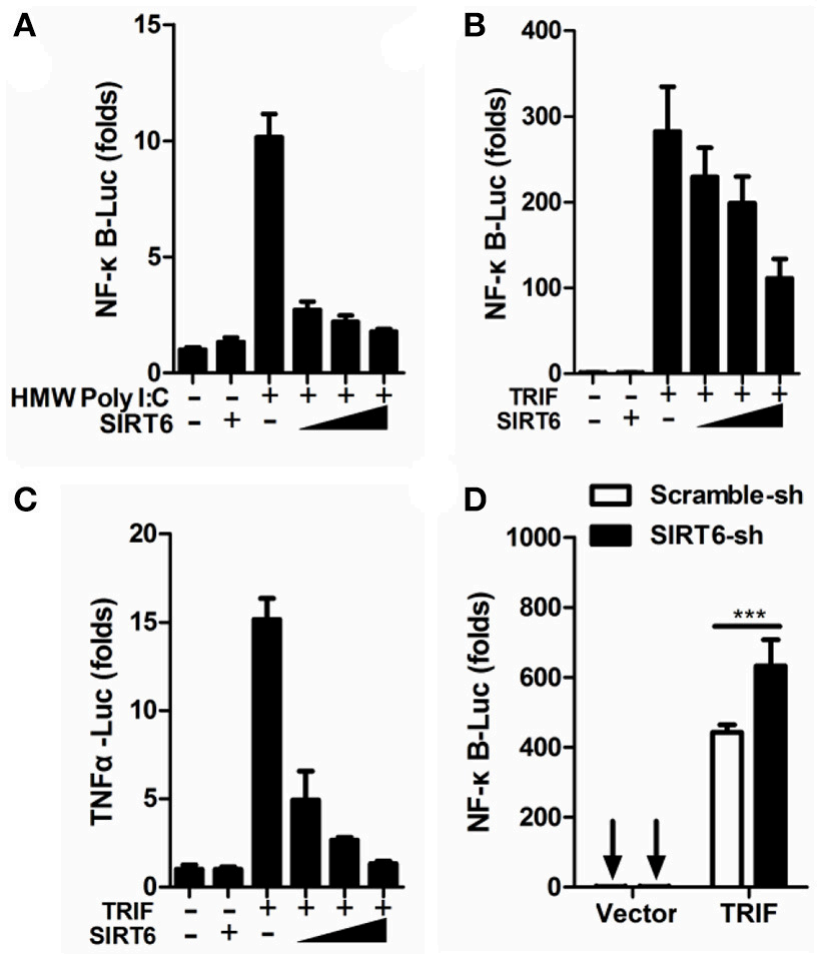
SIRT6 inhibits TLR3-mediated inflammatory response. **(A)** Quantification of NF-κB promoter activity of HEK293T-TLR3 cells transfected with either an empty vector or increasing amounts of SIRT6 plasmid and treated with HMW Poly I:C. Quantification of **(B)** NF-κB promoter activity and **(C)** TNFα promoter activity of HEK293T cells expressing TRIF together with increasing amounts of SIRT6. **(D)** Quantification of NF-κB promoter activity in scrambled shRNA or *SIRT6* shRNA treated HEK293T cells transfected with either an empty vector or TRIF plasmid. Data shown are the mean ± SEM; ^***^p < 0.001. Representative results are from at least three independent experiments.

In addition to RIG-I/MDA5, the Toll-like receptor 3 (TLR3) also play an important role in DENV recognition and induction of cytokine (Tsai et al., 2009). When we assessed whether SIRT6 regulated inflammatory responses via TLR3 pathway, we found that SIRT6 overexpression inhibited the activation of NF-κB promoter in HEK293T-TLR3 cells treated with extracellular Poly I:C (HMW) (Figure 3A). Further, when we co-expressed the TLR3 signaling adaptor, TRIF, with increasing amounts of SIRT6, SIRT6 significantly decreased the TRIF-induced NF-κB and TNFα promoter activation in a dose-dependent manner (Figures 3B–C). Furthermore, silencing of *SIRT6* promoted TRIF-induced NF-κB activation (Figure 3D) and Poly I:C (HMW) induced *Il6* production (Figure S3B). Taken together, these findings suggest that SIRT6 blocks TLR3-mediated inflammatory responses.

### NF-κB p65 is the target of SIRT6

**Figure 4:**
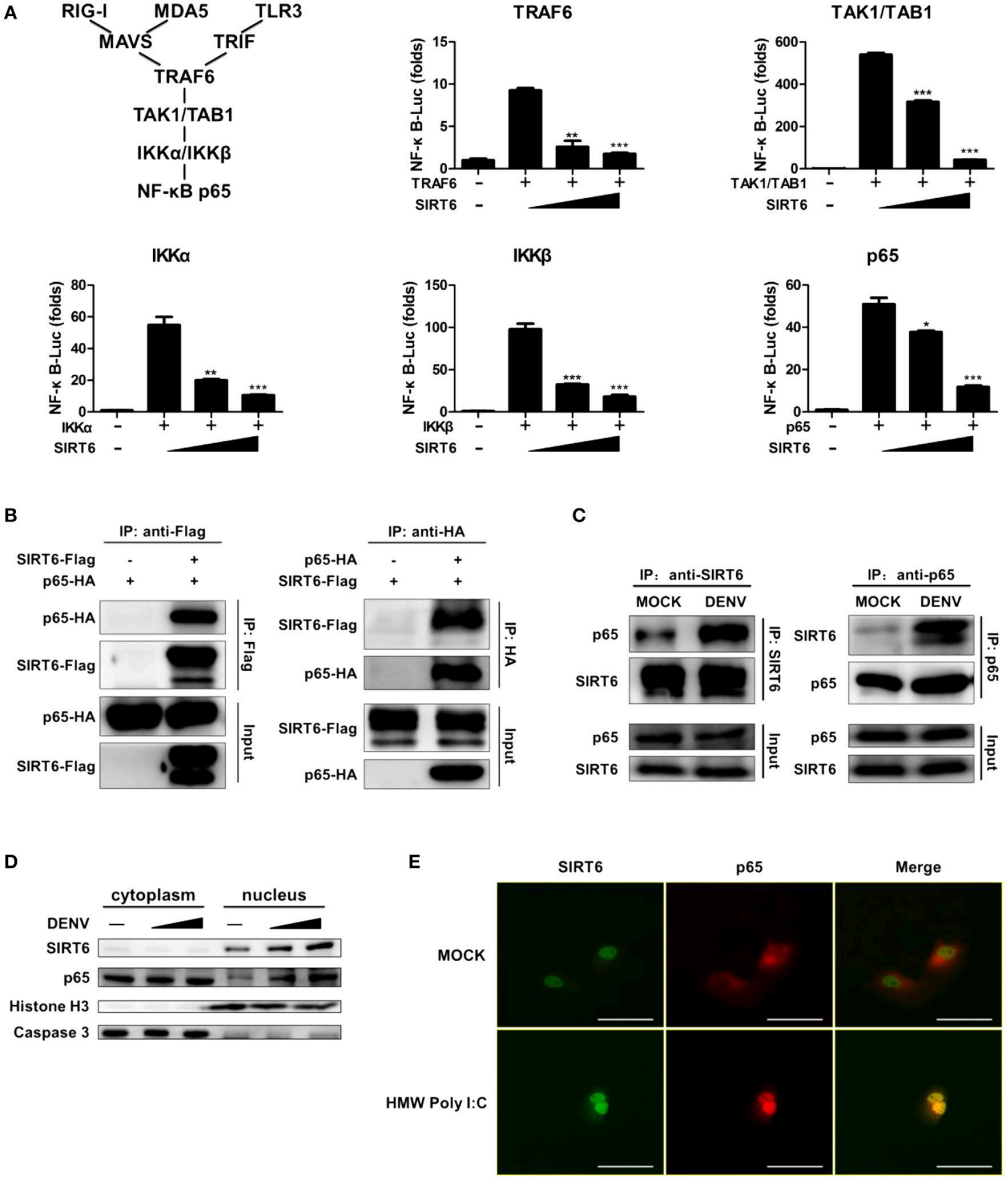
NF-κB p65 is the target of SIRT6. **(A)** SIRT6 inhibits RLR and TLR3-activated NF-κB signaling pathway. Quantification of NF-κB promoter activity of HEK293T cells expressing increasing amounts of SIRT6 together with TRAF6, TAK1/TAB1, IKKα, IKKβ, or p65, respectively. **(B)** SIRT6 interacts with NF-κB p65. Coimmunoprecipitation (CoIP) of SIRT6 and p65 from HEK293T cells expressing Flag-SIRT6 and HA-p65 using α-Flag or α-HA antibody pull-down, followed by immunoblotting. **(C)** CoIP of endogenous SIRT6 and p65 from Raw264.7 cells infected with DENV (MOI = 1) for 12 h using α-SIRT6 or α-p65 antibody pull-down, followed by immunoblotting. **(D)** Immunoblot analysis of SIRT6, p65, Caspase 3 and Histone H3 in cytoplasm and nucleus of Raw264.7 cells after DENV infection (MOI = 1–5) for 12 h. **(E)** Immunofluorescence microscopy of HEK293T-TLR3 cells transfected with plasmids of Flag-SIRT6 (green) with HA-p65 (red) stimulated by HMW Poly I:C. White Bar: 50 um. Data shown are the mean ± SEM; ^*^p < 0.05, ^**^p < 0.01, ^***^p < 0.001. Representative results are from at least three independent experiments.

To identify the target of SIRT6 in RLR and TLR3 signaling, we expressed TRAF6, TAK1/TAB1, IKKα, IKKβ, NF-κB p65 along with increasing amounts of SIRT6. The reporter assay showed that SIRT6 inhibited NF-κB activation induced by all these signaling molecules (Figure 4A). These data suggested that SIRT6 might target the downstream component, NF-κB p65. Then, we determined whether SIRT6 targets p65 using coimmunoprecipitation (CoIP) assays. SIRT6 plasmid with Flag tag and p65 plasmid with HA tag were transfected into HEK293T cells. The coimmunoprecipitation results showed that SIRT6 associated with p65 (Figure 4B). To confirm whether SIRT6 physically interacts with the NF-κB subunit p65, we immunoprecipitated endogenous SIRT6 and immunoblot analysis revealed a specific interaction with p65. In addition, SIRT6 was detected in IPs of endogenous p65. Importantly, DENV infection promoted endogenous SIRT6 binding to p65 in Raw264.7 cells (Figure 4C). We also immunoprecipitated endogenous NF-κB subunit p50 to investigate whether SIRT6 associates with the p50. We did not detect the interaction between p50 and SIRT6 (Figure S4).

To further verify that SIRT6 associated with p65, we performed subcellular fractionation and immunofluorescence microscopy. Endogenous p65 was mainly in the cytosol of untreated cells, but it translocated to nucleus and localized together with SIRT6 after DENV infection. Increased expression of SIRT6 was detected in the nuclear fraction upon DENV infection of Raw264.7 cells (Figure 4D). Consistent with these results, immunofluorescence microscopy demonstrated the colocalization of SIRT6 and p65 after Poly I:C treatment (Figure 4E). Taken together, these findings suggest that SIRT6 interacts with p65 in the nucleus in a viral infection-inducible manner.

### The core domain of SIRT6 is required for p65 inhibition

**Figure 5:**
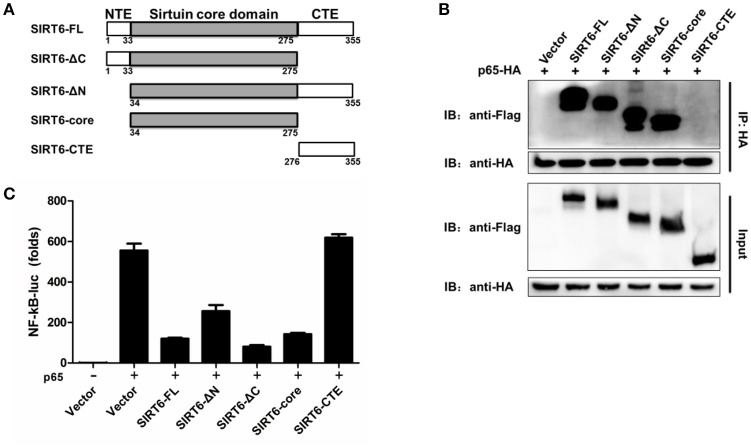
The core domain of SIRT6 is required for p65 inhibition. **(A)** Schematic of SIRT6 deletion mutants. **(B)** Coimmunoprecipitation of NF-κB p65 and SIRT6 from HEK293T cells expressing HA-p65 together with Flag-SIRT6 variants using a HA monoclonal antibody, followed by immunoblotting. **(C)** Quantification of NF-κB promotor activity of HEK293T cells transfected with either an empty vector or SIRT6 deletion mutants together with p65 plasmid.

We next asked which domain of SIRT6 binds to and inhibits p65 function. To determine which domain of SIRT6 was necessary for interaction with p65, we generated several SIRT6 deletion constructs (Figure 5A) and assessed them in coimmunoprecipitation experiments. NF-κB p65 bound strongly to full-length SIRT6, sirtuin core domain (amino acids 34–274), ΔN (amino acids 1–33 was deleted) and ΔC (amino acids 275–355 was deleted) constructs of SIRT6, while C terminus of SIRT6 lost the ability to interact with p65 (Figure 5B). These results suggest the core domain of SIRT6 is necessary for p65 binding. In addition, deletion of the N-terminal and C-terminal domain did not affect the inhibitory activity of SIRT6 on p65-induced NF-κB promoter activation. The core domain of SIRT6 retained a similar inhibitory effect on p65-induced NF-κB promoter activation as full-length SIRT6 (Figure 5C). These data suggest that the core domain of SIRT6 is required for p65 binding and inhibition.

### SIRT6 targets to the DNA binding domain of p65

**Figure 6:**
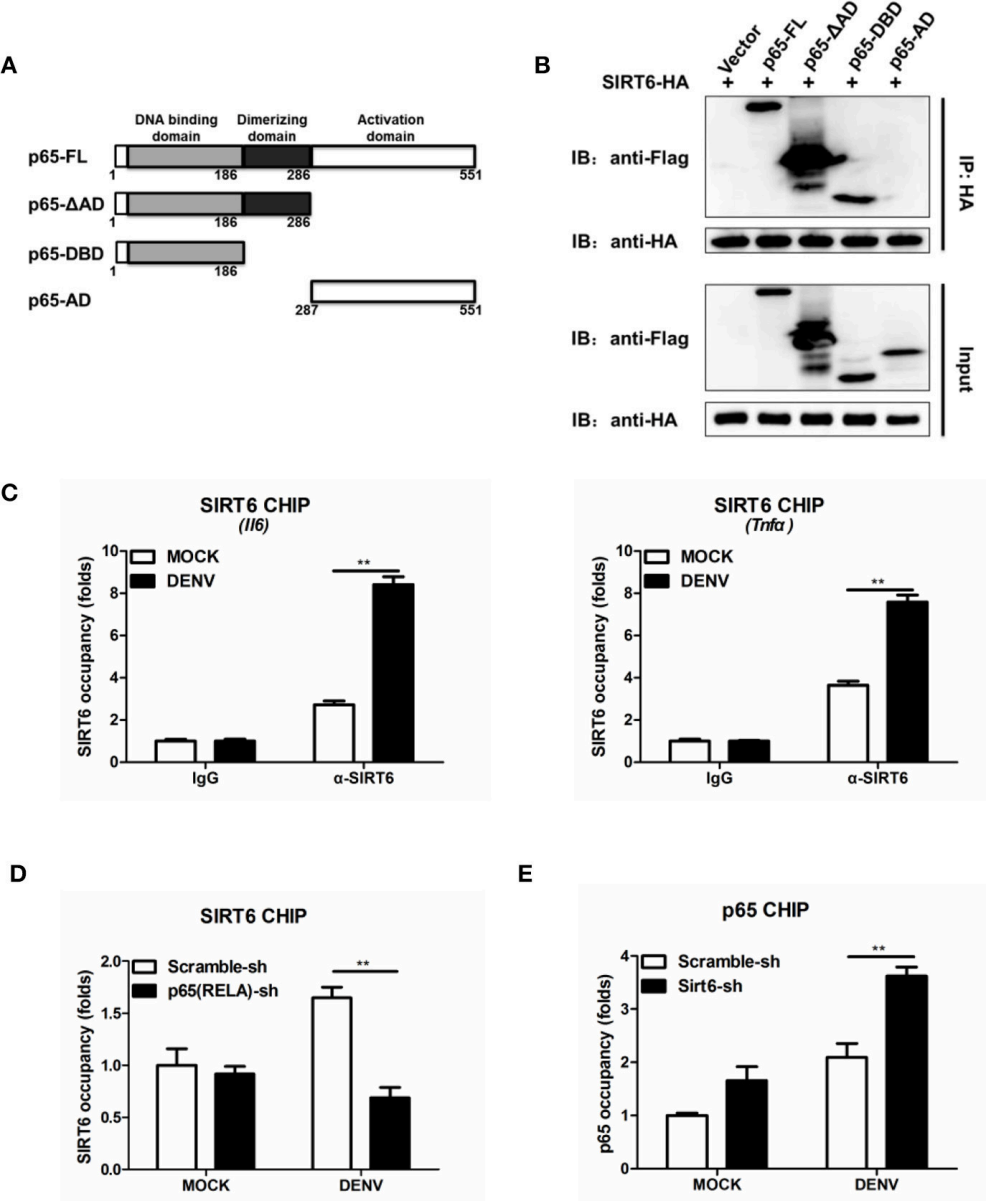
SIRT6 interacts with the DNA binding domain of p65 and decreases p65 occupancy in the *Il6* promoter. **(A)** Schematic of p65 deletion mutants. **(B)** Coimmunoprecipitation of SIRT6 and NF-κB p65 from HEK293T cells expressing HA-SIRT6 and Flag-p65 variants using α-HA monoclonal antibody, followed by immunoblotting. **(C)** Chromatin immunoprecipitation (ChIP) assays followed by qPCR for SIRT6 in the *Il6* and *Tnfa* promoter in Raw264.7 cells infected with DENV (MOI = 1) for 12 h. **(D)** ChIP assays followed by qPCR for SIRT6 in the *Il6* promoter in Raw264.7 cells stably expressing either scrambled shRNA or *p65*-trageting shRNA after DENV infection (MOI = 1) for 12 h. **(E)** ChIP assays followed by qPCR for NF-κB p65 in the *Il6* promoter in Raw264.7 cells stably expressing either scrambled shRNA or *Sirt6*-trageting shRNA after DENV infection (MOI = 1) for 12 h. Data shown are the mean ± SEM; ^**^p < 0.01. Representative results are from at least three independent experiments.

NF-κB p65 is composed of a DNA binding domain (amino acids 1–186), a dimerizing domain (amino acids 187–286) and an activation domain (amino acids 287–551) (Figure 6A). To identify the SIRT6-binding domain within p65, we performed coimmunoprecipitation and assessed the binding of SIRT6 to several truncated forms of p65. We found that SIRT6 bound strongly to full-length, ΔAD and DNA binding domain of p65, but failed to bind to the activation domain of p65 (Figure 6B). Together these data suggest that SIRT6 may inhibit NF-κB activation through targeting the DNA binding domain of p65.

### SIRT6 decreases p65 occupancy in the *Il6* promoter

In response to viral infections, activated NF-κB translocates to the nucleus, binds to the promoter region of target genes, and induces the expression of inflammatory cytokines (Rahman and McFadden, 2011). To test whether SIRT6 occupies the p65 target gene promoter region, we performed chromatin immunoprecipitation (ChIP) assays with an anti-SIRT6 antibody. SIRT6 was detected at the promoter of p65 target gene *Il6 and Tnfa*, compared to the IgG negative control background signal. Notably, DENV infection further elevated the binding of SIRT6 to the *Il6 and Tnfa* promoter (Figure 6C). These data suggest that SIRT6 is enriched at the promoter region of p65 regulated genes following viral infection. To determine whether SIRT6 is recruited to p65 target gene promoter region via its interaction with p65, we designed shRNA that targeted *p65* and generated *p65*-silenced Raw264.7 cells. Silencing of p65 significantly decreased the occupancy of SIRT6 at *Il6* gene promoter (Figure 6D). Finally, we examined the effects of SIRT6 deficiency on p65 occupancy at target gene promoters by ChIP assay using anti-p65 antibody. In *Sirt6*-silenced cells, p65 occupancy at *Il6* gene promoter was significantly enhanced after DENV infection (Figure 6E). These data suggest that SIRT6 is recruited to the inflammatory gene promoter through its interaction with the DNA binding domain of p65, and impairs the binding of p65 to its target gene promoter.

## Discussion

Excessive inflammation is believed to play a pathologic role during host response to DENV infection (Costa et al., 2013). Increased levels of proinflammatory cytokines and chemokines occur in patients experiencing severe dengue disease (Chaturvedi et al., 2000; Soundravally et al., 2014). Therefore, elucidating the regulatory mechanisms in the DENV-induced host immune responses is a key task. Sirtuins may play an important role in the control of inflammation. In this study, we found that mRNA and protein levels of SIRT6 increased upon DENV infection. It has been reported that transcription factor AP-1 activation is induced in DENV-infected macrophages (Ceballos-Olvera et al., 2010). SIRT6 is a direct transcriptional target of AP-1 (Min et al., 2012). The DENV-induced activation of AP-1 may promote SIRT6 expression. We further investigated the role of SIRT6 in DENV-triggered immune responses. Silencing of *Sirt6* by RNA interference enhanced DENV-induced proinflammatory cytokine and chemokine production. Activation of the host innate immune system by DENV is thought to induce a strong inflammatory response. We next confirmed SIRT6 as a negative regulator in the RLR and TLR3-mediated innate immune response. Overexpression of SIRT6 significantly inhibited RIG-I/MDA5 and TLR3-induced NF-κB activation, whereas silencing of *SIRT6* had opposite effects on NF-κB activation. These findings suggest that SIRT6 has a global role in RLR and TLR signaling pathways, indicating its importance in virus induced innate immune responses.

NF-κB is a key component in the pathway from pathogen recognition to inflammatory cytokine production. NF-κB activation must be tightly regulated to prevent excessive immune responses. A20, CUEDC2, and ANGPTL8 have been identified as regulators that limit NF-κB signaling through different molecular mechanisms. A20 is a deubiquitinating enzyme that removes ubiquitin moieties from the signaling molecule TRAF6, thereby terminating TLR-induced NF-κB activation (Boone et al., 2004). CUEDC2 has been reported to interact with IKKα and IKKβ and inhibit NF-κB activation by decreasing phosphorylation of IKK (Li et al., 2008). ANGPTL8 has been reported to negatively regulate NF-κB activation by facilitating p62-mediated autophagic degradation of IKKγ (Zhang et al., 2017). In this study, several lines of evidence indicate SIRT6 directly targets NF-κB p65 to inhibit NF-κB activation. First, overexpression of SIRT6 inhibits either p65-induced or upstream factors of RLR/TLR3-signaling-induced NF-κB promoter activation. Second, p65 colocalizes with SIRT6 in the nucleus after infection with DENV. Third, DENV infection promotes endogenous SIRT6 binding to p65. Domain mapping assays show that the sirtuin core domain of SIRT6 is required for the binding of p65. Our results demonstrate that SIRT6 core domain alone is sufficient for the inhibitory effect of SIRT6 on NF-κB signaling.

Our findings and those of others suggest that sirtuins could target NF-κB p65 and regulate its function through distinct mechanisms. SIRT1 was initially reported to interact with p65 and deacetylate p65 at lysine 310 (Yeung et al., 2004). Overexpression of SIRT1 and the addition of the SIRT1 agonist resveratrol protects against microglia-dependent amyloid-β toxicity through inhibiting NF-κB signaling (Chen et al., 2005). Similar studies have revealed that SIRT2 interacts with p65 in the cytoplasm and deacetylates p65 *in vitro* and *in vivo* at lysine 310 (Rothgiesser et al., 2010). *Sirt2* deficient mice are more sensitive to DSS-induced colitis compared to wild type littermates (Lo et al., 2014). Surprisingly, SIRT5 and SIRT1/2 have opposite functions. SIRT5 could compete with SIRT2 to interact with p65 and block the deacetylation of p65 by SIRT2, leading to increased acetylation of p65 and the activation of NF-κB signaling (Qin et al., 2017). SIRT6 has been shown previously to affect NF-κB-dependent gene expression, through deacetylation of histone H3 (Kawahara et al., 2009). Our study has demonstrated SIRT6 interacts with the DNA binding domain of p65. SIRT6 is recruited to cytokines promoter regions through its interaction with p65 after DENV infection. This interaction decreased the occupancy of p65 at the NF-κB target gene promoter. Consequently, SIRT6 occupies the promoter of NF-κB target genes and mediates repression of inflammatory response.

In summary, our work identifies a critical role for SIRT6 in the control of DENV-induced innate immune responses. Mechanistically, SIRT6 targets the DNA binding domain of NF-κB p65 and blocks the NF-κB medicated transcription of inflammatory cytokines. SIRT6 may be an effective target for new therapeutic interventions against various infectious and inflammatory diseases that have been associated excessive inflammation.

## Author contributions

FQia and PL designed the study. PL, YJ, and FQi performed the experiments. FW, SL, and YC provided experimental assistance. PL, FQia, and RM interpreted the data. PL and FQia drafted the manuscript. All the authors reviewed the manuscript.

### Conflict of interest statement

The authors declare that the research was conducted in the absence of any commercial or financial relationships that could be construed as a potential conflict of interest.

## Supplementary material

Figure S1 Effect of SIRT6 on DENV replication. Quantification of intracellular DENV loads by qPCR from HEK293T cells stably expressing either scrambled shRNA or *SIRT6*-targeting shRNA after DENV infection for 36 h. Data shown are the mean ± SEM; ^***^*p* < 0.001.

Figure S2 Effect of SIRT6 on ΔRIG-I, MDA5 and MAVS expression. Quantification of ΔRIG-I **(A)**, MDA5 **(B)**, and MAVS **(C)** protein expression levels by immunoblotting from HEK293T cells transfected with either an empty vector or indicated plasmids, together with increasing amounts of SIRT6.

Figure S3 SIRT6 inhibits RLR- and TLR3-mediated inflammatory response. Quantification of *Il6* mRNA levels by qPCR from Raw264.7 cells stably expressing either scrambled shRNA or *Sirt6*-targeting shRNA after SeV infection **(A)** and PolyI:C (HMW) stimulation **(B)** for 12 h. Data shown are the mean ± SEM; ^*^*p* < 0.05, ^**^*p* < 0.01.

Figure S4 SIRT6 does not associate with NF-κB subunit p50. **(A)** Coimmunoprecipitation of SIRT6 and p65 from HEK293T cells infected with DENV (MOI = 1) for 24 h using α-p65 antibody pull-down, followed by immunoblotting using α-SIRT6 antibody. **(B)** Coimmunoprecipitation of SIRT6 and p50 from HEK293T cells infected with DENV (MOI = 1) for 24 h using α-p50 antibody pull-down, followed by immunoblotting using α-SIRT6 antibody.
