# Supplementary figures and images for: SIRT6 Acts as a Negative Regulator in Dengue Virus-Induced Inflammatory Response by Targeting the DNA Binding Domain of NF-κB p65
Source: Front Cell Infect Microbiol. 2018 Apr 9;8:113. doi: 10.3389/fcimb.2018.00113 (PMC5900784; doi:10.3389/fcimb.2018.00113)

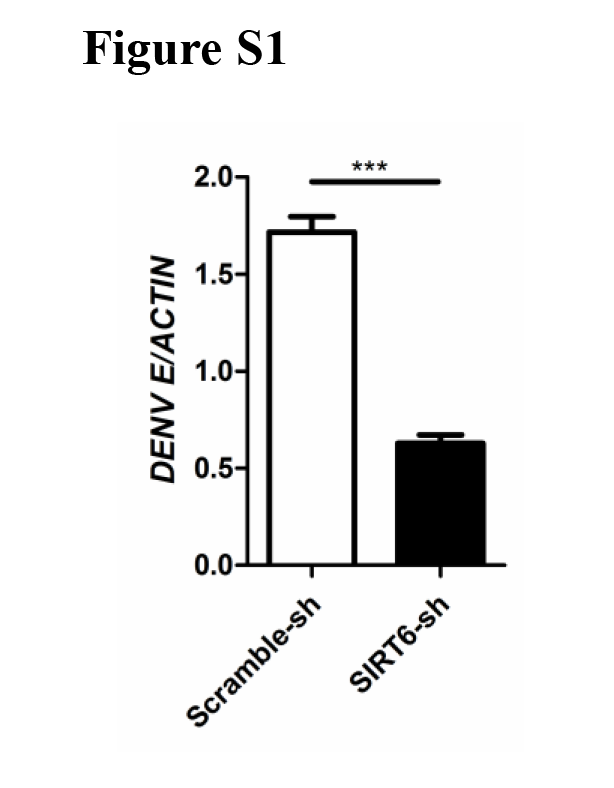

Supplement: Figure S1 — Effect of SIRT6 on DENV replication. Quantification of intracellular DENV loads by qPCR from HEK293T cells stably expressing either scrambled shRNA or SIRT6-targeting shRNA after DENV infection for 36 h. Data shown are the mean ± SEM; ***p < 0.001. [file Image1.TIF]

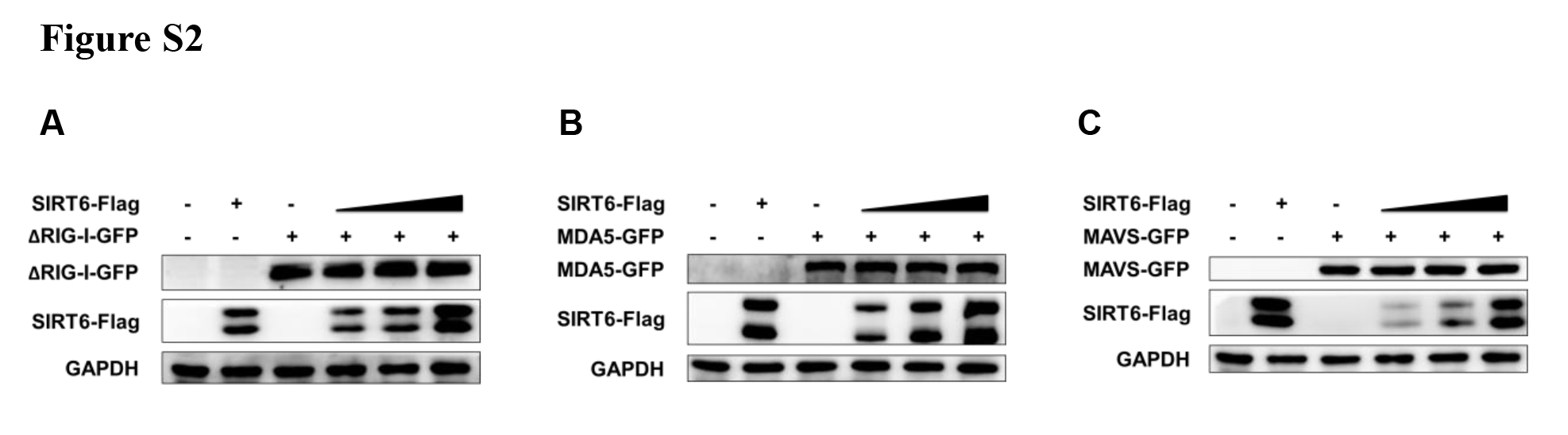

Supplement: Figure S2 — Effect of SIRT6 on ΔRIG-I, MDA5 and MAVS expression. Quantification of ΔRIG-I (A), MDA5 (B), and MAVS (C) protein expression levels by immunoblotting from HEK293T cells transfected with either an empty vector or indicated plasmids, together with increasing amounts of SIRT6. [file Image2.TIF]

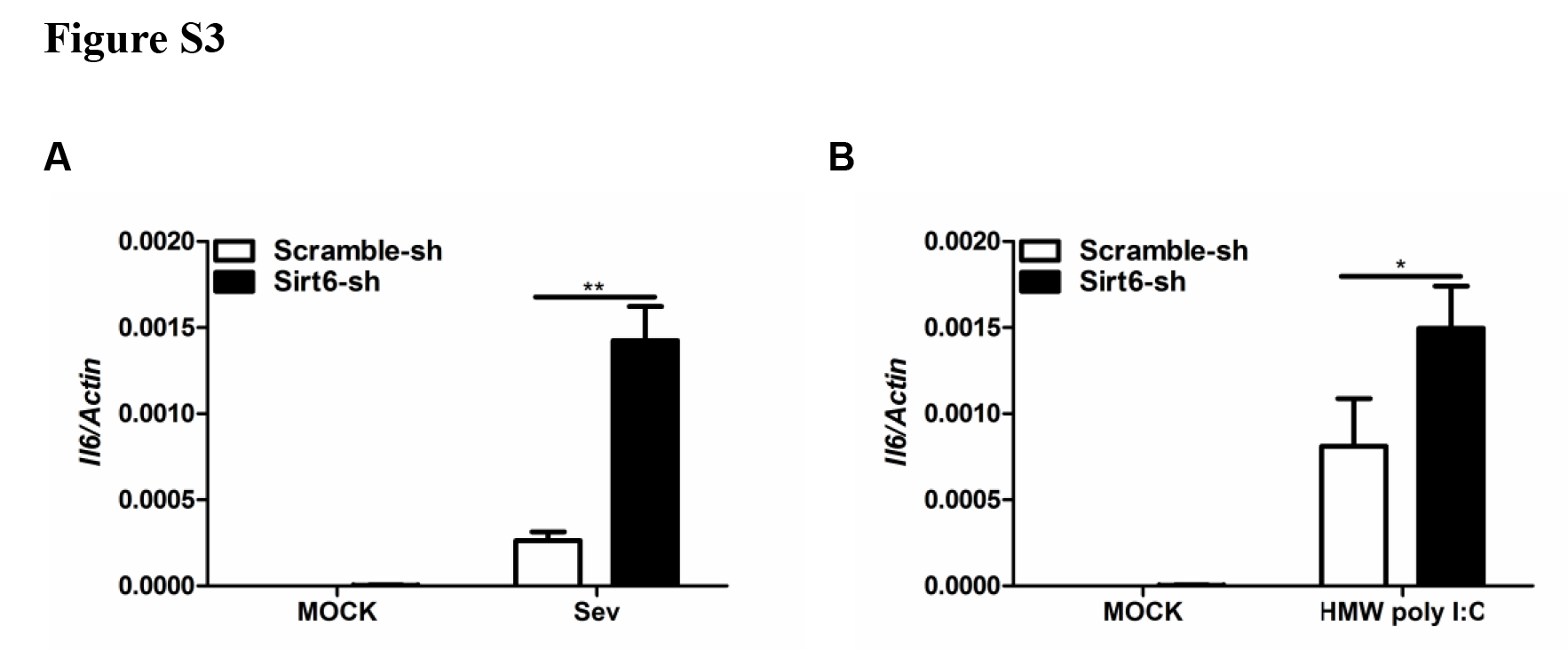

Supplement: Figure S3 — SIRT6 inhibits RLR- and TLR3-mediated inflammatory response. Quantification of Il6 mRNA levels by qPCR from Raw264.7 cells stably expressing either scrambled shRNA or Sirt6-targeting shRNA after SeV infection (A) and PolyI:C (HMW) stimulation (B) for 12 h. Data shown are the mean ± SEM; *p < 0.05, **p < 0.01. [file Image3.TIF]

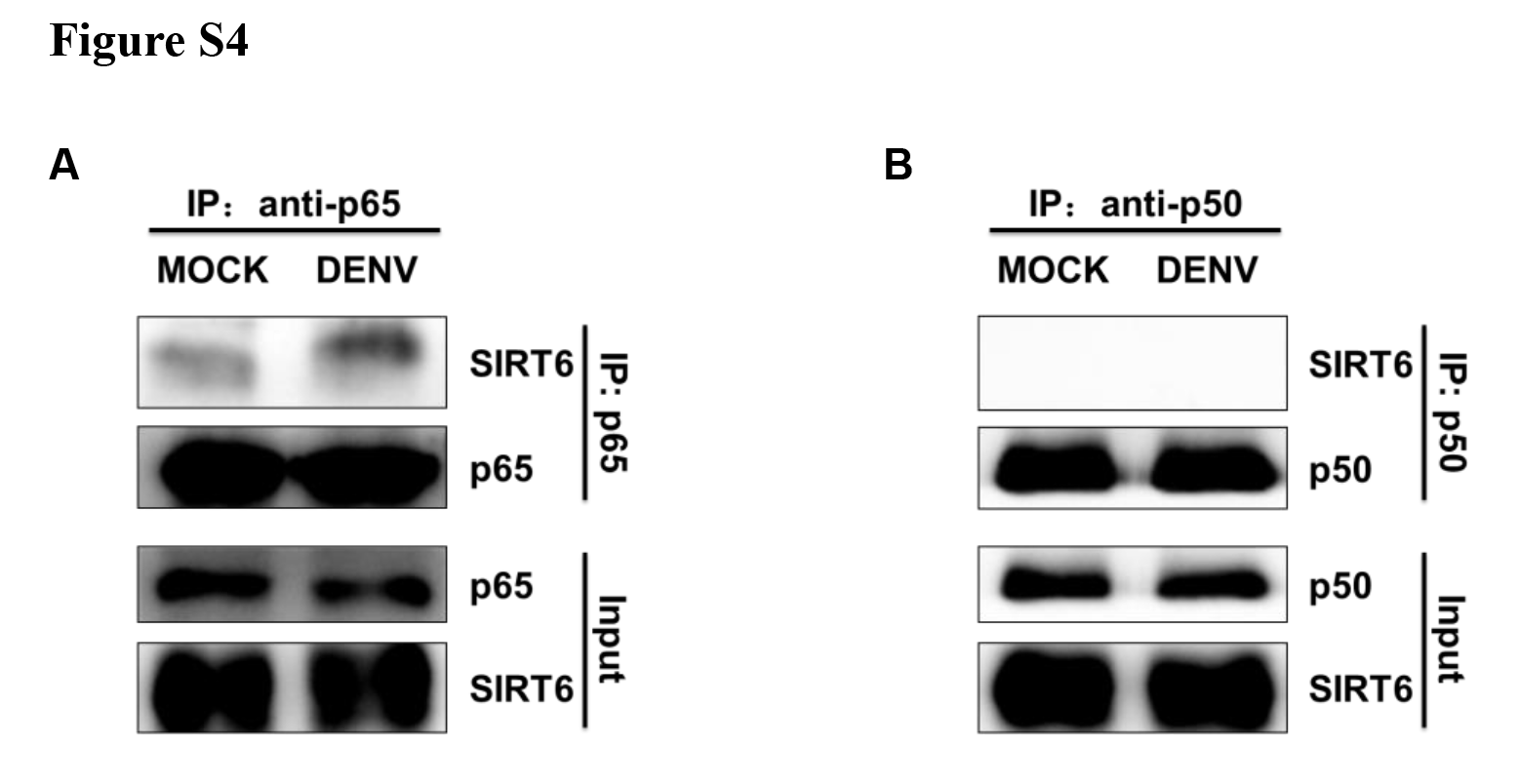

Supplement: Figure S4 — SIRT6 does not associate with NF-κB subunit p50. (A) Coimmunoprecipitation of SIRT6 and p65 from HEK293T cells infected with DENV (MOI = 1) for 24 h using α-p65 antibody pull-down, followed by immunoblotting using α-SIRT6 antibody. (B) Coimmunoprecipitation of SIRT6 and p50 from HEK293T cells infected with DENV (MOI = 1) for 24 h using α-p50 antibody pull-down, followed by immunoblotting using α-SIRT6 antibody. [file Image4.TIF]
